# Six new species of *Cymatodera* from Mexico and Central America and the retention of *Cymatodera obliquefasciata* as a valid name (Cleridae, Tillinae)

**DOI:** 10.3897/zookeys.299.4359

**Published:** 2013-05-14

**Authors:** Alan F. Burke

**Affiliations:** 1 Department of Entomology, 123 West Waters Hall, Kansas State University, Manhattan, KS 66506-4004, USA

**Keywords:** Coleoptera, *Cymatodera*, *Bogcia*, New World, taxonomy, antennae, genitalia, terminalia

## Abstract

Six new *Cymatodera* speciesfrom the Mexican states of Jalisco and Chiapas, and the Central American countries of El Salvador, Honduras, Costa Rica and Panamá are described: *Cymatodera rosalinae* **sp. n.**, *Cymatodera capax* **sp. n.**, *Cymatodera sinuosa* **sp. n.**, *Cymatodera vittata* **sp. n.**, *Cymatodera rubida* **sp. n.** and *Cymatodera limatula* **sp. n.** Justification for retaining *Cymatodera obliquefasciata* within *Cymatodera* instead of transferring it to *Bogcia* is provided. Male genitalia and other characters of taxonomic value are illustrated.

## Abstract (es)

Se describen seis nuevas especies del género *Cymatodera* pertenecientes a los estados Mexicanos de Jalisco y Chiapas, y los países Centroamericanos de El Salvador, Honduras, Costa Rica y Panamá: *Cymatodera rosalinae* **sp. n.**, *Cymatodera capax* **sp. n.**, *Cymatodera sinuosa* **sp. n.**, *Cymatodera vittata* **sp. n.**, *Cymatodera rubida* **sp. n.** y *Cymatodera limatula* **sp. n.** Se proporciona información sobre la retención de *Cymatodera obliquefasciata* dentro de *Cymatodera*, invalidando así su transferencia a *Bogcia*. Se presentan ilustraciones de la genitalia masculina así como otros caracteres de valor taxonómico.

## Introduction

The subfamily Tillinae Leach, 1835 has a worldwide distribution with 543 described species classified in 67 genera (Opitz 2010). The fauna is well represented in the North American temperate and subtemperate zones, the Neotropics, and the Paleotropic region of Africa, with an important concentration of genera and species in Madagascar (Opitz 2010). In the New World, the subfamily comprises the following genera: *Araeodontia* Barr, 1952, *Barrotillus* Rifkind, 1996, *Bogcia* Barr, 1978, *Bostrichoclerus* Van Dyke, 1938, *Callotillus* Wolcott, 1911, *Cylidrus* Latreille, 1825, *Cymatodera* Gray, 1832, *Cymatoderella* Barr, 1962, *Lecontella* Wolcott and Chapin, 1918, *Monophylla* Spinola, 1841 and *Onychotillus* Chapin, 1945. The Tillinae is a taxonomically complex group. Polymorphism is frequently encountered, the boundaries between both genera and species are rather difficult to establish, and it is common to observe species that appear to bridge the gap between generic taxa.

Barr (1948, 1952a, 1952b, 1972, 1978) and Rifkind (1993, 1995, 1996) are responsible for most of the recent descriptions and classificatory work treating the Tillinae of Mexico and Central America with a focus mainly on the fauna of northern mainland Mexico and Baja California. Despite some progress, most of the central and southern portion of Mexico has been poorly surveyed. Furthermore, our understanding of the Central American Tillinae fauna is even more limited, and our taxonomic knowledge of this geographical region has been scarcely added to in almost a century.

*Cymatodera* is the most speciose Tillinae genus in the New World, with a distribution that extends from southern Canada to Colombia and Venezuela (Opitz 2010). The highest diversity of described species is found in the southwest portion of the United States and the north of Mexico (Barr, personal communication). *Cymatodera* is well represented in Mexico (Rifkind 1993). Vaurie (1952) noted the presence of 37 species inhabiting the country, although the records she presented were mostly limited to north-central Mexico. At present, 20 species have been described from Central America (Barr, unpublished checklist).

The genus *Bogcia* was erected by Barr (1978) and is represented by two species, *Bogcia oaxacae* Barr and *Bogcia disjuncta* Barr, both restricted to the western portion of Mexico. Members of this group show strong similarities to *Cymatodera*; however, theycan be reliably separated based on the structure of the protarsal unguis. The position of the tarsal claw is in close approximation with the denticle in *Bogcia* (Fig. 22), rather than conspicuously separated as in *Cymatodera* (Fig. 23). Barr (unpublished checklist) included *Cymatodera obliquefasciata* Schaeffer within *Bogcia*. This placement was based on the strongly serrate condition of the antennae (Fig. 16). Nevertheless, I have found that this trait is not reliable for distinguishing *Bogcia* from *Cymatodera*. The antenna of the latter is highly variable, and does not represent a character that marks a clear discontinuity in the group. Consequently, it is possible to encounter antennal forms that range from filiform (Fig. 18), as in *Cymatodera longicornis* LeConte, or moderately serrate (Fig. 19), such as *Cymatodera antennata* Schaeffer, to strongly serrate (Fig. 15), as observed in the new species, *Cymatodera limatula*, described below. Therefore, *Cymatodera obliquefasciata* should be retained as *Cymatodera* based on the ungual structure of the protarsus.

This work is intended to be a small contribution towards a better understanding of the *Cymatodera* fauna of Mexico and Central America, and to shed some light on the complex relationships found in this group.

## Methods

The methods used for genitalia extraction and dissection are similar to those described by Ekis (1977). Terminology used partially follows the work of Ekis (1977) and Rifkind (1996). I considered differences in the aedeagi as the primary evidence for determination of biological species (*sensu* Mayr 1963).

Specimens were observed using a Leica MZ APO stereomicroscope. All measurements were made using a stereomicroscope ocular micrometer and the software Leica Application Suite V. 3.4.0. Optic images were taken using a Leica DFC 500 digital camera. Scanning electron photographs were taken using a Hitachi 3500N variable pressure scanning electron microscope.

The following abbreviations are used in the description of the holotypes: **TL**= Total body length, **HW**= Maximum head width, **HL**= Head length, **PW**= Maximum pronotal width, **PL**= Pronotal length, **EW**= Maxium elytral width, **EL**= Elytral length.

### Acronyms of collections used here in are:

**CNIN** Colección Nacional de Insectos, Instituto de Biología, UNAM, México

**FSCA** Florida State Collection of Arthropods, Gainesville, FL, USA

**INBC** Instituto Nacional de Biodiversidad, Colección Entomológica, Santo Domingo de Heredia, Costa Rica

**JEWC** James E. Wappes Collection, San Antonio, TX, USA

**JNRC** Jacques Rifkind Collection, Valley Village, CA, USA

**KSUC** Kansas State University Museum of Entomological and Prairie Arthropod Research, Kansas State University, Manhattan, KS, USA

**RGCG** Roland Gerstmeier Collection, Technische Universitat Munchen, Freising, Germany

**RHTC** Robert H. Turnbow Jr. Collection, Enterprise, AL, USA

**SEMC** Snow Entomological Collection, University of Kansas, Lawrence, KS, USA

**TAMU** Texas A&M Insect Collection, Texas A&M University, College Station, TX, USA

**USNM** United States National Museum of Natural History, Smithsonian Institution, Washington D.C., USA

**WFBM** William F. Barr Museum, University of Idaho, Moscow, ID, USA

**WOPC** Weston Opitz Collection, Salina , KS, USA

## Taxonomy

### Genus Cymatodera

Gray, 1832

http://species-id.net/wiki/Cymatodera

Cymatodera See Barr (1952a), Barr (1972) and Vaurie (1952) for references and keys to species.

#### Type species.

*Cymatodera hopei* Gray, 1832: 375.

#### Diagnosis.

Members of the genus *Cymatodera* may be separated from other genera of the New World Tillinae by the following combination of characters (partially adopted from Opitz 2002): 1) frons simple, without prominent horns, 2) elytra with striated punctuations, 3) antenna strongly serrate to filiform, 4) last antennomere circular in cross section, shorter than the length of preceding three antennomeres, 5) tarsal pulvillar formula 4-4-4, 6) tibial spur formula: 2-2-2, 6) basal denticle of tarsal claws trigonal, 7) eyes coarsely faceted, 8) body length 4.0 - 30.0 mm.

### Cymatodera rosalinae

Burke sp. n.

urn:lsid:zoobank.org:act:4F6DAD66-F4F6-4772-A0D5-94AE0492488C

http://species-id.net/wiki/Cymatodera_rosalinae

Figs 1 , 9, 10 , 20 , 24, 31 , 38, 45 , 52

#### Type material.

**Holotype:** male, México, Jalisco, Estación Biológica Chamela UNAM, 80 m, 19-VII-1993, J. E. Wappes, red handwritten label, holotype deposited USNM. **Paratypes:** 27 males, 25 females. 11 males and 11 females: same data as holotype (CNIN, 6; JEWC, 6; KSUC, 2; RGCG, 4; TAMU, 4); 1 male and 2 females: México, Jalisco, Costa Careyes, at light, tropical deciduous forest, 7-VII-1991, J. Rifkind and P. Gum (JNRC, 3); 1 female: México, Jalisco, carretera no. 200, Costa Careyes, blacklight, tropical deciduous forest, 6-VII-1993, J. & E. Beierl (TAMU, 1); 6 males and 4 females: México, Jalisco, Estación Biológica Chamela UNAM, 9 to 19-VII-1993, J. Huether (FSCA, 2; CNIN, 2; TAMU, 2; WOPC, 3; JNRC, 1); 8 males and 5 females: México, Jalisco, municipio de La Huerta, Estación Biológica Chamela UNAM, 9 to 19-VII-1993, Morris, Huether and Wappes (RHTC, 4; JNRC, 4; USNM, 4; WOPC, 1); 1 male and 1 female: México, Jalisco, Estación Biológica Chamela, 3 to 4-VIII-1994, R. L. Westcott (WFBC, 2); 1 female: México, Jalisco, Chamela, 7-VII-1990, F. A. Noguera (CNIN, 1).

#### Description.

**Figures 1–8.**
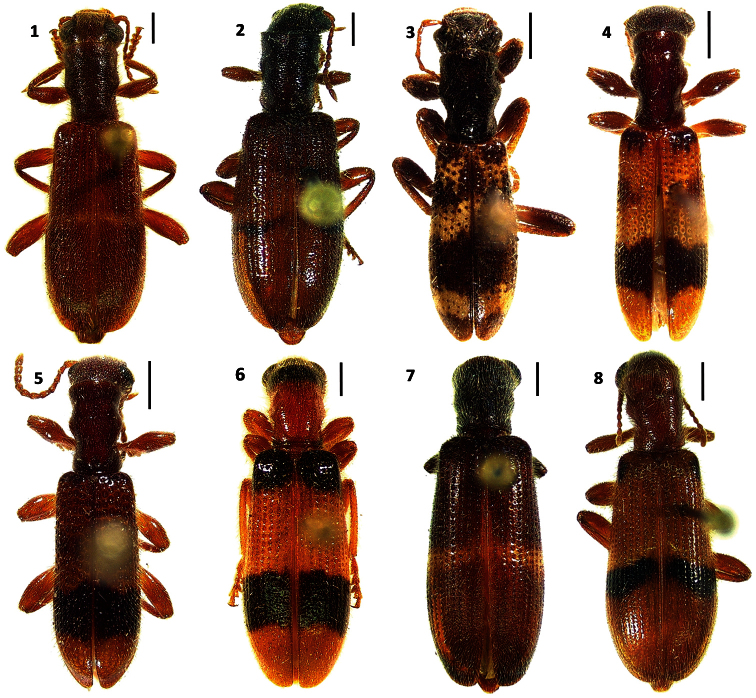
Habitus of: **1** *Cymatodera rosalinae* sp. n. (holotype male) **2** *Cymatodera capax* sp. n. (holotype female) **3** *Cymatodera sinuosa* sp. n. (holotype female) **4** *Cymatodera vittata* sp. n. (holotype male) **5** *Cymatodera rubida* sp. n. (holotype male) **6** *Cymatodera limatula* sp. n. (holotype male) **7** *Cymatodera obliquefasciata* Schaeffer **8** *Bogcia oaxacae* Barr. Scale bars = 1 mm.

Size: TL= 12.5 mm, length of males: 8.8 - 13.5 mm, length of females 9.8 - 15.2 mm, n = 53 (Fig. 1).

Color: head and pronotum fuscous-brown; rest of the body uniformly brown. Each elytron with a pair of median, slightly oblique, pale fascia that extends from elytral suture to epipleuron.

**Figures 9–17.**
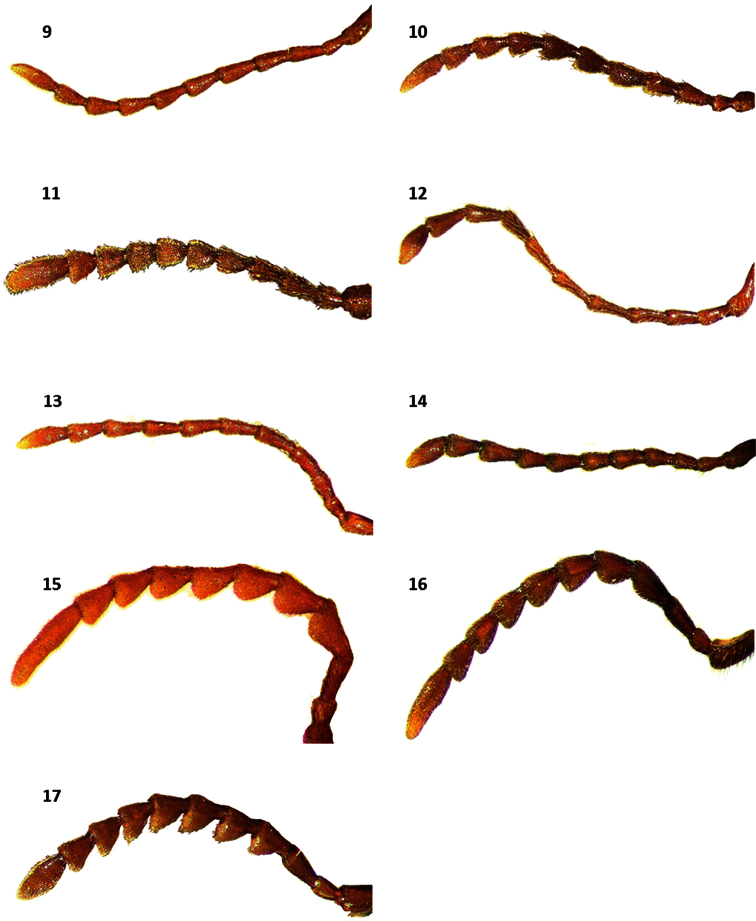
Antennae of: **9** *Cymatodera rosalinae* (male) **10** *Cymatodera rosalinae* (female) **11** *Cymatodera capax* (male) **12** *Cymatodera sinuosa* (male) **13** *Cymatodera vittata* (male) **14** *Cymatodera rubida* (male) **15** *Cymatodera limatula* (male) **16** *Cymatodera obliquefasciata* (male) **17** *Bogcia oaxacae* (male).

Head: HL= 2.1 mm, HW= 1.3 mm; length to width ratio: males average 1.68, females average 1.55; measured across eyes wider than pronotum; finely, rather punctate; somewhat clothed with short, recumbent setae intermixed with less numerous, erect setae; surface rugose, except frons shiny. Eyes rather big, somewhat rounded, inconspicuously longer than wide, emarginate in front, bulging laterally, separated by approximately 2.5 eye-widths (Fig. 20). Antennae extending to base of elytra; third antennomere 2.0 × longer than preceding antennomere; antennomeres 3-10 subequal in length; antennomeres 2-4 slender; antennomeres 5-10 feebly serrate; last antennomere elongate, subacuminate, 1.5 × longer than preceding antennomere (Fig. 9).

Thorax: PL= 3.2 mm, PW= 1.9 mm; length to width ratio: males average 1.56, females average 1.62; pronotum widest at middle, middle slightly broader than anterior margin; sides constricted subapically, more strongly constricted behind middle; disc flat, indistinctly impressed in front of middle; clothed with short, recumbent setae intermixed with long, erect and suberect setae; surface somewhat rugose; slightly more densely punctate than head; subbasal tumescences indistinctly pronounced. Mesosternum scarcely, coarsely punctate. Metasternum convex, puncticulate; covered with fine, recumbent setae. Scutellum subquadrate, notched posteriorly, covered with short, erect setae.

Legs: vested with short, recumbent setae intermixed with long, suberect setae that become more densely arranged on proximal face of tibiae; femora rugulose, moderately, finely punctate; tibiae transversely rugose, moderately, coarsely punctate, vested with short, recumbent setae intermixed with occasional semierect setae.

Elytra: EL= 7.6 mm, EW= 3.4 mm; length to width ratio: males average 2.25, females average 1.78; anterior margin bisinuate; humeri rounded; sides subparallel; base wider than pronotum; widest behind middle; disc flattened apically; apices sinuate, feebly dehiscent; surface feebly rugose; vestiture composed of short, semirecumbent setae intermixed with less numerous, long, erect and semierect setae; sculpturing consisting of small, coarse punctations arranged in striae that gradually reduce in size behind middle; interstices smooth, 4.0 × the width of punctation.

Abdomen: ventrites 1-5 rugulose, vested with short, recumbent setae and some long, semierect setae. First visible ventrite indistinctly, finely punctate; ventrites 2-5 densely, finely punctate. Fifth visible ventrite convex; lateral margins oblique; posterior margin broadly, rather deeply, semicircularly emarginate (Fig. 24). Sixth visible ventrite subquadrate; surface somewhat excavated medially, convex laterally; rather punctate; rugulose; covered with short recumbent setae; lateral margins oblique; posterior margin broadly, moderately deeply, arcuately emarginate; hind angles rounded (Fig. 24). Fifth tergite slightly convex; finely punctate; rugulose; posterior margin broadly, shallowly, arcuately emarginate (Fig. 38). Sixth tergite subrectangular; rugulose; surface convex; broader than long; moderately, finely punctate; inconspicuously covered with short, recumbent setae; lateral margins oblique; posterior margin broadly, shallowly emarginate; hind angles arcuate (Fig. 38). Posterior margin of sixth tergite fully covering sixth visible ventrite. Aedeagus 2.25 mm long, robust, ratio of length of paramere to whole tegmen 0.28: 1; parameres rather prominent, subparallel, pointed distally, phallobase wide; phallus with copulatory piece tapered at apex, phallic plate with a row of large, sclerotized denticles along dorsal margin; phallobasic apodeme and endophallic struts broad, somewhat dilated distally (Fig. 52).

**Figures 18–21.**
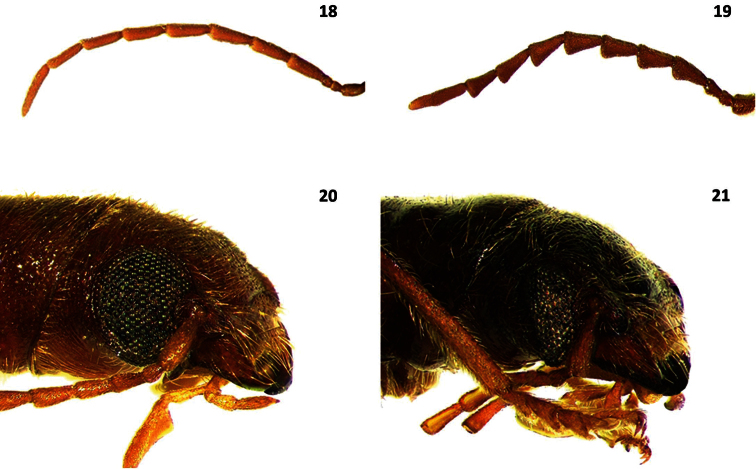
Antennae and head in lateral view of: **18** *Cymatodera longicornis* (male) **19** *Cymatodera antennata* (male) **20** *Cymatodera rosalinae* **21** *Cymatodera sinuosa*.

**Figures 22–23.**
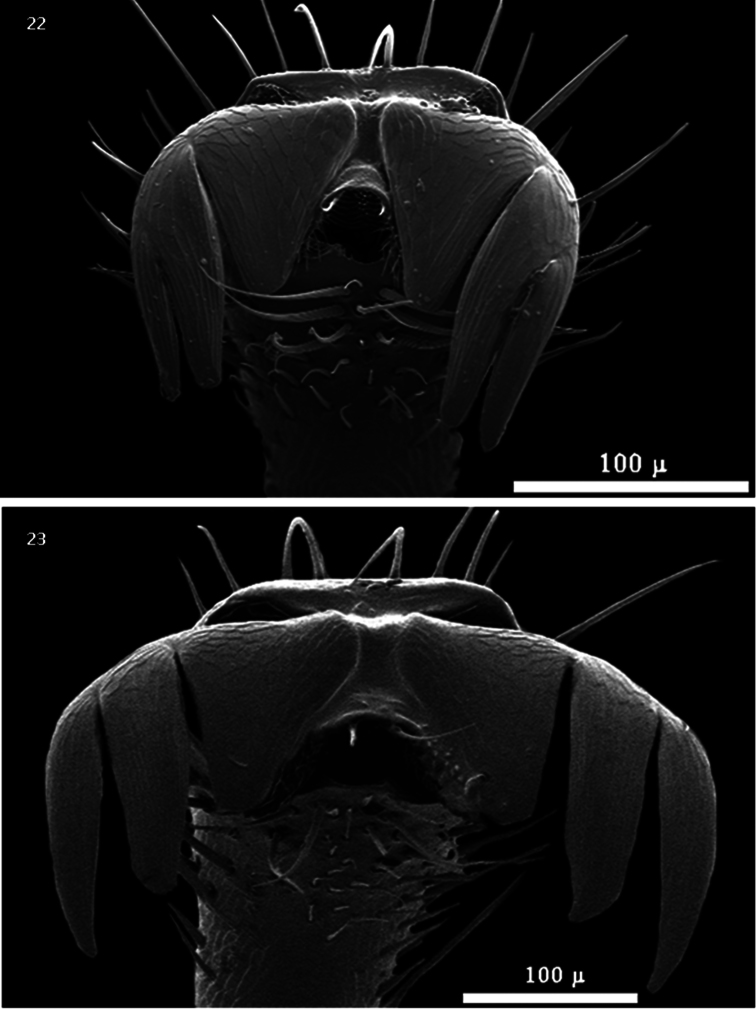
Ungual structure of the protarsi of: **22** *Bogcia oaxacae* **23** *Cymatodera obliquefasciata*.

**Figures 24–37.**
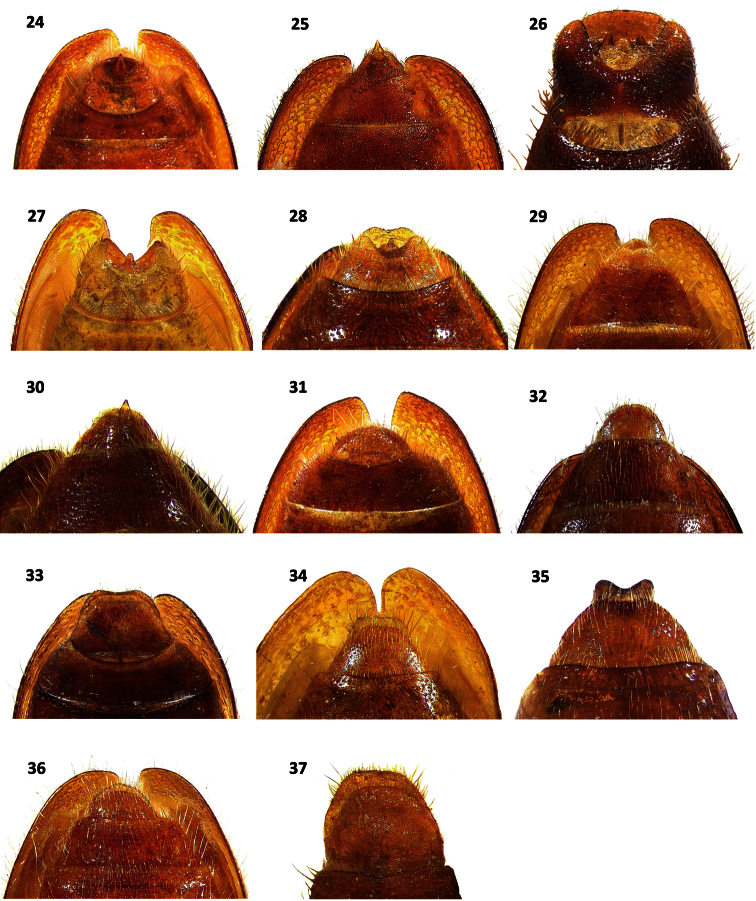
Terminalia in ventral view of: **24** *Cymatodera rosalinae* (male) **25** *Cymatodera capax* (male) **26** *Cymatodera sinuosa* (male) **27** *Cymatodera vittata* (male) **28** *Cymatodera rubida* (male) **29** *Cymatodera limatula* (male) **30** *Cymatodera obliquefasciata* (male) **31** *Cymatodera rosalinae* (female) **32** *Cymatodera capax* (female) **33** *Cymatodera sinuosa* (female) **34** *Cymatodera vittata* (female) **35** *Cymatodera rubida* (female) **36** *Cymatodera limatula* (female) **37** *Cymatodera obliquefasciata* (female).

**Figures 38–51.**
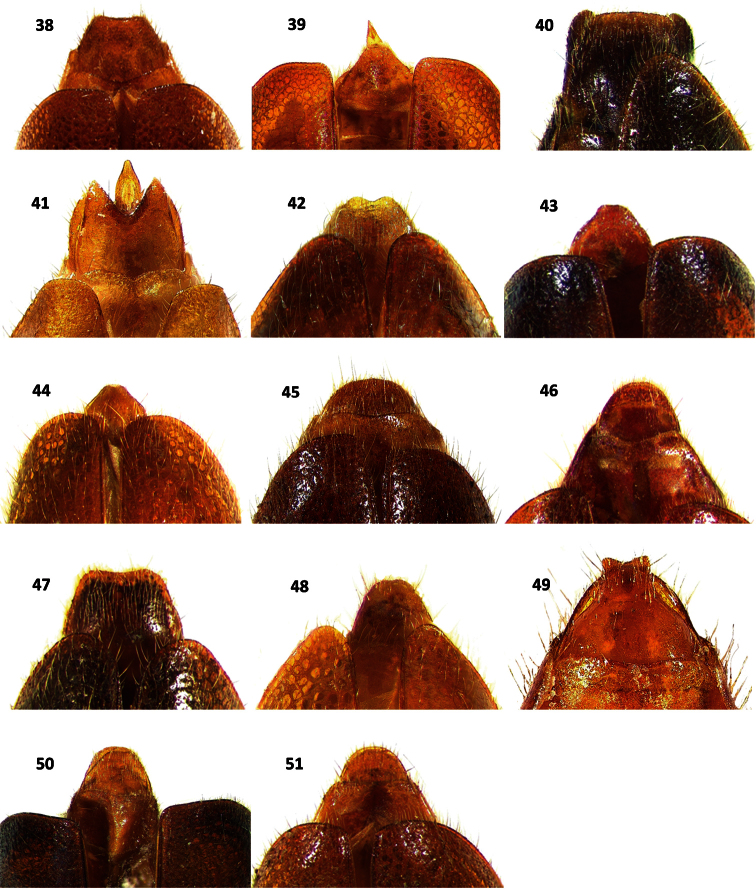
Terminalia in dorsal view of: **38** *Cymatodera rosalinae* (male) **39** *Cymatodera capax* (male) **40** *Cymatodera sinuosa* (male) **41** *Cymatodera vittata* (male) **42** *Cymatodera rubida* (male) **43** *Cymatodera obliquefasciata* (male) **44** *Cymatodera oaxacae* (male) **45** *Cymatodera rosalinae* (female) **46** *Cymatodera capax* (female) **47** *Cymatodera sinuosa* (female) **48** *Cymatodera vittata* (female) **49** *Cymatodera rubida* (female) **50** *Cymatodera obliquefasciata* (female) **51** *Bogcia oaxacae* (female).

**Figures 52–57.**
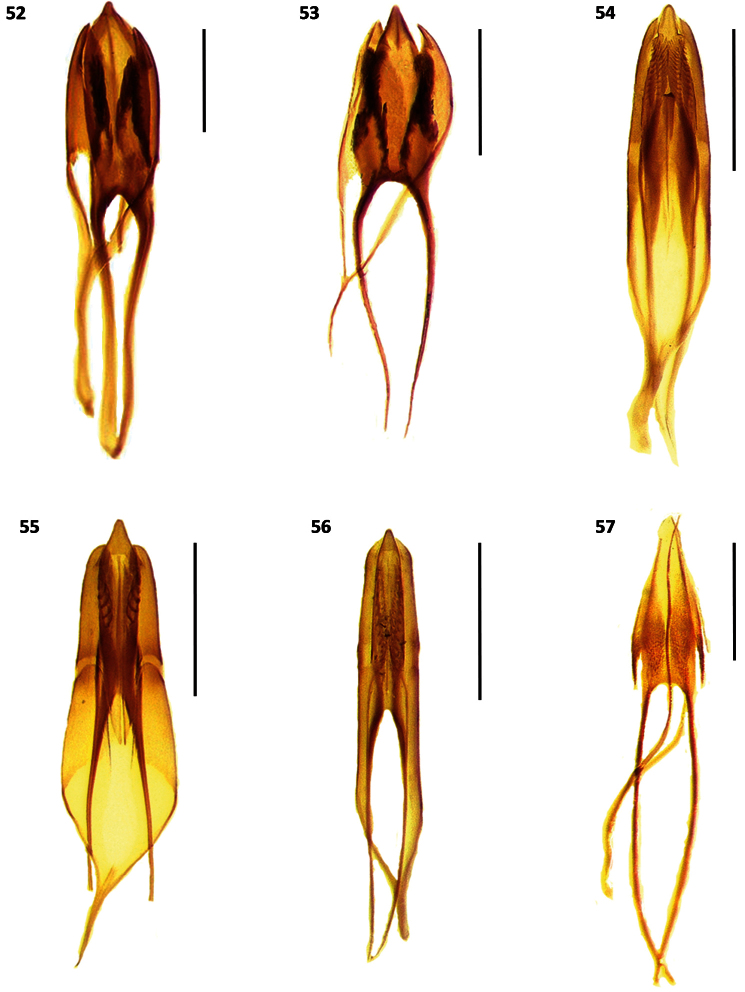
Male genitalia of: **52** *Cymatodera rosalinae* **53** *Cymatodera capax* **54** *Cymatodera sinuosa* **55** *Cymatodera vittata* **56** *Cymatodera rubida* **57** *Cymatodera limatula*. Scale bars = 500 µ.

#### Variation.

Female specimens differ from males in the following respects: antennal serration somewhat more evident (Fig. 10); elytral margins less parallel, rendering a somewhat more robust appearance; posterior margin of fifth visible ventrite broadly, shallowly,arcuately emarginate (Fig. 31); sixth visible ventrite subtriangular, lateral margins strongly oblique, almost confluent apically, posterior margin rather acuminate (Fig. 31); posterior margin of fifth tergite narrowly, moderately emarginate (Fig. 45); sixth tergite semicircular, lateral and posterior margins broadly rounded (Fig. 45); sixth tergite fully covering sixth visible ventrite. Pronotum and elytral ground color rather inconsistent in both sexes, ranging from testaceous to fuscous-brown. Midelytral fascia variably marked, almost imperceptible in some specimens. Some individuals possess an irregular, midelytral black band ranging in size from a complete fascia, to a pair of inconspicuous maculae on each side of elytra.

#### Differential diagnosis.

Distinguishable from congeners based on its size, antennal shape, midelytral marking, terminal abdominal segments and male genitalia. This species appears most similar to the allopatric species *Cymatodera obliquefasciata*. Color, form, elytral sculpturing, median pale fascia and serrate condition of antennae are characters shared by *Cymatodera rosalinae* (Fig. 1) and *Cymatodera obliquefasciata* (Fig. 7). This new species can be separated from the latter based on the following respects: *Cymatodera rosalinae* has the antennomeres 2-4 slender, antennomeres 5-10 transversally robust in dorsal view and somewhat serrate (Fig. 9), due to sexual dimorphism, serrate condition of female specimens is somewhat more evident (Fig. 10), last antennomere internally sinuate, and humeral maculae absent. *Cymatodera obliquefasciata* has the antennomeres 1-3 slender, antennomeres 4-10 transversally slender in dorsal view and boldly serrate (Fig. 16), and humeral maculae variably marked to absent. In addition, the moderately incised and arcuately emarginate posterior margin of the sixth visible ventrite in male specimens of *Cymatodera rosalinae* (Fig. 24) is absent in males of *Cymatodera obliquefasciata* (Fig. 30). The female of *Cymatodera rosalinae* (Figs 31, 45) can be distinguished from females of *Cymatodera obliquefasciata* (Fig. 37, 50) by the distinctive shape of its abdominal terminalia.

#### Distribution.

Known from the vicinity of the Chamela Biological Station, situated in the Chamela-Cuixmala region, on the western portion of Jalisco, Mexico.

#### Etymology.

I am very pleased to name this new species in honor of my mother, Rosalina Roco, a cornerstone in my life, and a person whose endless efforts have been of inspirational support during my professional career.

### Cymatodera capax

Burke sp. n.

urn:lsid:zoobank.org:act:195F1972-989D-4F78-A4FF-5197CBE754B9

http://species-id.net/wiki/Cymatodera_capax

Figs 2 , 11 , 25, 32 , 39, 46 , 53

#### Type material.

**Holotype:** female, Costa Rica, Provincia de Guanacaste, Playa Naranjo, Parque Nacional Santa Rosa, 350 m, (10°50.94'N, 85°36.69'W), XII-1990, E. Alcázar, “INBIO CRI000486511”, red handwritten label, holotype deposited in INBC. **Paratypes:** 8 males, 5 females. 4 males and 4 females: same data as holotype, except 1 female collected I-1991 (INBC, 3; RGCG, 1; JNRC, 2; KSUC, 2); 1 male: Costa Rica, Provincia de Guanacaste, Estación Las Pailas, Parque Nacional Rincón de la Vieja, 800 m, 18-XII-1993, F. A. Quesada (WOPC, 1); 1 male: Costa Rica, Provincia de Guanacaste, Estación Las Pailas, Parque Nacional Rincón de la Vieja, 10 to 27-III-1993, K. Taylor (WFBC, 1); 1 male: Costa Rica, Provincia Guanacaste, Sector Las Pailas, 800 m, (10°47.38’ N, 85°18.69’ W), 16 to 30-III-1995, K. Taylor (USNM, 1); 1 female: Costa Rica, Provincia de Guanacaste, Estación Las Pailas, Parque Nacional Rincón de la Vieja, 800 m, 1-IV-1991, D. Fernández (CNIN, 1); 1 male: Costa Rica, Provincia Guanacaste, Las Pailas, Parque Nacional Rincón de la Vieja, 10 to 20-IV-1994, D. García (WOPC, 1).

#### Description.

Size: TL= 11.3 mm, length of males 8.5 - 11.25 mm, length of female 10.5 - 11.5 mm, n= 14 (Fig. 2).

Color: head and pronotum dark brown; rest of the body uniformly brown. Each elytron with a post median, irregular, narrow, obliquely directed, black fascia that extends from epipleuron to elytral suture, becoming somewhat paler near suture; this fascia is bordered anteriorly by an inconspicuous pale marking; ventrites 1-5 with a pair of irregular, testaceous maculae near sides.

Head: HL= 2.2 mm, HW 1.95 mm; length to width ratio: males average 1.14, females average 1.22; measured across eyes wider than pronotum; finely punctate; surface rugose; vested with short, recumbent setae intermixed with few long, semirecumbent setae that become more numerous toward epistoma. Eyes medium-sized, rather rounded, inconspicuously longer than wide, emarginate in front, somewhat bulging laterally, separated by approximately 3 eye-widths. Antennae extending to base of elytra; third antennomere 2.0 × the length of second

antennomere; fourth antennomere slightly shorter than third antennomere; antennomeres 4-10 subequal in length; antennomeres 2-4 subcylindrical; antennomere 5-10 gradually becoming serrate toward distal end; last antennomere irregularly elongate, sinuate internally, 1.5 × longer than tenth antennomere (Fig. 11).

Thorax: PL= 2.7 mm, PW= 1.9 mm; length to width ratio: males average 1.36, females average 1.44; anterior and posterior margins of pronotum as wide as middle; sides feebly constricted subapically; slightly more constricted behind middle; disc flat, inconspicuously impressed in front of middle; moderately, coarsely punctate; less densely punctate than head; surface rugose; vested with short, recumbent setae, intermixed with long erect setae; less densely clothed than head; subbasal tumescences moderately projected. Mesosternum clothed with long, recumbent setae; coarsely punctate. Metasternum smooth, convex; rather puncticulate. Scutellum semicircular, covered with short, recumbent setae, posterior margin slightly notched.

Legs: somewhat covered with short and long semirecumbent setae that become more numerous on second half of tibiae; femora finely punctate, longitudinally rugose; tibiae coarsely punctate, transversely rugose.

Elytra: EL= 6.2 mm, EW= 3.45 mm; length to width ratio: males average 1.61, females average 1.76; anterior margin bisinuate, broader than pronotum; humeri rounded; sides subparallel; widest behind middle; disc subflattened above; surface rugose; apices rounded, feebly dehiscent; clothed with short, semirecumbent setae intermingled with less densely, longer, erect setae; sculpturing consisting on coarse punctations arranged in striae that gradually reduce in size behind middle; interstices 3.0 × the width of punctation.

Abdomen: ventrites 1-5 rugulose; inconspicuously vested with short, recumbent setae; sparsely, finely punctate. Fifth visible ventrite convex; lateral margins oblique; posterior margin truncate (Fig. 32). Sixth visible ventrite semicircular; rugulose; broader than long; surface feebly convex; moderately, finely punctate; lateral and posterior margins broadly rounded (Fig. 32).

Fifth tergite rugulose; surface convex; lateral margins oblique; posterior margin broadly, shallowly, arcuately emarginate; posterior angles rounded (Fig. 46). Sixth tergite subtriangular; broader than long; surface feebly convex; rugulose; moderately, finely punctate; lateral margins oblique; posterior margin broadly, semicircularly rounded (Fig. 46). Posterior margin extending slightly beyond apical projection of sixth visible ventrite. Aedeagus 1.85 mm long; conspicuously robust, ratio of length of parameres to whole tegmen 0.31: 1; parameres prominent, conspicuously pointed at apex, subtriangular; phallobase wide; phallus with copulatory piece tapered distally, phallic plate with a row of long, prominent denticles along dorsal margin; phallobasic apodeme and endophallic struts slender (Fig. 53).

#### Variation.

Male specimens differ from females by having the posterior margin of the fifth visible ventrite broadly, shallowly, arcuately emarginate (Fig. 25); sixth visible ventrite subtriangular, rugulose, surface slightly convex, broader than long, lateral margins rather oblique, posterior margin broadly, shallowly, triangularly emarginated, hind angles arcuate (Fig. 25); posterior margin of fifth tergite broadly, shallowly emarginate (Fig. 39); sixth tergite subtriangular, rugulose, surface convex, as broad as long, lateral margins strongly oblique, posterior margin broadly rounded (Fig. 39); sixth tergite extending slightly beyond apical margin of sixth visible ventrite. Midelytral fascia variably marked in both sexes, ranging from strongly to feebly impressed. Leg color is also rather inconsistent, ranging from uniformly brown to bicolored.

#### Differential diagnosis.

Separable from other *Cymatodera* species based on its shape, elytral marking, unique terminal abdominal segments and male genitalia (Fig. 53). Due to the serrate condition of the antennae (Fig. 11), general size, form, color and midelytral fascia, *Cymatodera capax* (Fig. 2) appears closest to the Mexican *Bogcia oaxacae* (Fig. 8).The new species can be distinguished from the latter based on ungual differences of the protarsus. *Cymatodera capax* presents the claw of the protarsus conspicuously separated (Fig. 23) from the denticle, rather thanclosely approximated, as observed in *Bogcia oaxacae* (Fig. 22).

#### Distribution.

Known from two localities in the Guanacaste Province, Costa Rica: Playa Naranjo, adjacent to Santa Rosa, Guanacaste National Park; and Sector Las Pailas, Rincón de la Vieja National Park.

#### Etymology.

The specific epithet comes from the Latin word capax (= wide), a noun that makes allusion to the overall robust appearance of this new species.

### Cymatodera sinuosa

Burke sp. n.

urn:lsid:zoobank.org:act:D461C70D-9EA3-49C5-A270-65F53F19749F

http://species-id.net/wiki/Cymatodera_sinuosa

Figs 3 , 12 , 21 , 26, 33 , 40, 47 , 54

#### Type material.

**Holotype:** female, Honduras, Olancho, Parque Nacional La Muralla, 1350 m, 1-VI-1995, R. H. Turnbow, red handwritten label, holotype deposited in USNM. **Paratypes:** 2 males, 4 females. 3 females: same data as holotype, except 1 female collected 24 to 27-V-1995 (KSUC, 1; RHTC, 2); 2 males (1 male dissected, body not recovered); 1 female: El Salvador, Departamento de San Salvador, El Boquerón, 22-VI-1959, L. J. Bechyne (INBC, 1; WOPC, 1).

#### Description.

Size: TL= 10.4 mm, length of males 10.8 - 12.2 mm, length of females 7.75 - 10.5 mm, n = 7 (Fig. 3).

Color: head and pronotum fuscous; elytra, scutellum and legs brown, except posterior half of femora dark brown; antennae and mouthparts testaceous; abdomen, meso and metasternum light brown. Each elytron with three irregular, variably sinuate, pale fasciae; first on anterior fourth, slender, extending from elytral suture to epipleuron, surrounding scutellum; next fascia on second fourth, broader than preceding band, extending from elytral suture to epipleuron; third fascia on last fourth, slightly shorter and narrower than preceding fascia, extending from elytral suture to tenth stria, not reaching epipleuron.

Head: HL= 2.2 mm, HW= 1.95 mm; length to width ratio: males average 1.08, females average 1.17; measured across eyes wider than pronotum; densely, coarsely punctate; surface rugose; clothed with short, recumbent setae intermixed with long, erect setae; frons feebly bi-impressed. Eyes somewhat small, subsinuate, longer than wide, moderately emarginated in front, bulging laterally, separated by approximately 4 eye-widths (Fig. 21). Antennae slender; loosely composed; extending to basal sixth of elytra; antennomeres 2-3 subequal in length; fourth antennomere slightly longer than third antennomere; antennomeres 4-10 subequal in length; antennomeres 5-10 weakly serrate; last antennomere flattened apically (Fig. 12).

Thorax: PL= 2.5 mm, PW= 1.75 mm; length to width ratio: males average 1.45, females average 1.39; pronotum widest at middle, middle slightly broader than anterior margin; sides constricted subapically, more strongly constricted behind middle; disc flat, feebly impressed in front of middle; subbasal tumescences pronounced; surface rugose, moderately, finely punctate;

less densely punctate than head; covered with short, recumbent setae interspaced with less numerous, long, erect and suberect setae. Mesosternum coarsely punctate. Metasternum convex; puncticulate; surface rugulose; median impression strongly indicated. Scutellum semicircular, covered with some short, recumbent setae.

Legs: vested with short, recumbent setae intermixed with occasional long, suberect and erect setae that become more densely arranged on tibiae; femora rugulose; tibiae transversely rugose.

Elytra: EL= 5.5 mm, EW= 2.5 mm; length to width ratio: males average 2.26, females average 2.19; anterior margin arcuately emarginate, as wide as pronotum; humeri feebly indicated; sides subovoid, widest at second third; disc moderately flattened apically; apex broadly, separately rounded, dehiscent, covering sixth tergite; surface smooth, clothed with short, recumbent setae combined with less numerous, long, erect setae; sculpturing consisting on rather numerous, coarse punctations arranged in striae that abruptly reduce in size and become less numerous on last fourth; interstices about 2.0 × the width of punctation.

Abdomen: ventrites 1-5 rugose; each segment with a pair of large, shallow impressions near sides; somewhat clothed with short, fine, pale, recumbent setae; moderately, coarsely punctate. Fifth visible ventrite convex; lateral margins oblique; posterior margin broadly, deeply, arcuately emarginate (Fig. 33). Sixth visible ventrite subquadrate; rugulose; broader than long; surface convex; posterior half depressed at middle; puncticulate; lateral margins oblique; posterior margin truncate; hind angles rounded (Fig. 33). Fifth tergite rather convex; finely rugulose; lateral margins subparallel; posterior margin truncate. Sixth tergite subrectangular, broader than long; surface convex; rugulose; clothed with long, recumbent setae; scarcely, coarsely punctate; lateral margins oblique; posterior margin broadly, shallowly emarginate; hind angles arcuate (Fig. 47). Sixth tergite extending slightly beyond posterior margin of sixth visible ventrite. Aedeagus 2.0 mm long; ratio of length of paramere to whole tegmen 0.29: 1; parameres somewhat slender, elongate, subparallel, obtuse at apex, phallobase moderately broad; phallus with copulatory piece acuminated distally, phallic plate with numerous, long denticles along dorsal margin; phallobasic apodeme rather wide, not dilated distally; endophallic struts slender (Fig. 54).

#### Variation.

Males have the fifth visible ventrite convex, rugose, moderately, coarsely punctate, lateral margins oblique, posterior margin broadly, deeply, arcuately emarginate (Fig. 26); sixth visible ventrite rectangular, surface convex, rugose, moderately, coarsely punctate, with a longitudinal, median depression that extends from middle to posterior margin, lateral margins parallel, becoming somewhat oblique on last third, posterior margin broadly, deeply, arcuately incised, strongly elevated before emargination, forming a conspicuous ridge bordering the area preceding the posterior margin, then abruptly descending toward emargination, hind

angles robust, strongly arcuate at apex (Fig. 26); fifth tergite punctate, rugulose, posterior margin broadly, shallowly, arcuately emarginate; sixth tergite rectangular, longer than broad, rugulose, surface convex, moderately, finely punctate, lateral margins subparallel, posterior margin shallowly emarginate, finely crenulate (Fig. 40). Elytral ground color is slightly variable in both sexes, ranging from brown to fuscous. Leg color ranges from bicolored to uniformly brown. Fasciae color is also rather inconsistent, ranging from stramineous to testaceous. One paratype female displaysthe posterior margin of fifth tergite broadly, shallowly, arcuately emarginated.

#### Differential diagnosis.

This species recalls various *Cymatodera* members that are similar in color, fasciae pattern, body proportions and antennal *gestalt*. Among these, *Cymatodera sinuosa* is most similar to the Central American *Cymatodera parallela* Gorham, 1882 and the Mexican *Cymatodera grossa* Gorham, 1882. *Cymatodera sinuosa* can be separated from the former as follows: anterior margin of elytra as wide as pronotum (wider in *Cymatodera parallela*); elytral margins conspicuously wide behind second half (somewhat subparallel in *Cymatodera parallela*); elytral apices (Fig. 3) broadly rounded and dehiscent (moderately rounded and feebly confluent in *Cymatodera parallela*); third antennomere (Fig. 12) as long as preceding antennomere (1.5 × the length of preceding antennomere in *Cymatodera parallela*). Furthermore, this new species is distinguishable from *Cymatodera grossa* based on its unique pronotal and elytral sculpturing. *Cymatodera sinuosa* has the pronotum finely, moderately punctate (coarsely, densely punctate, with punctations nearly confluent in *Cymatodera grossa*); pronotal disc conspicuously impressed in front of middle (flat in *Cymatodera grossa*); antescutelar impression strongly indicated (inconspicuously indicated in *Cymatodera grossa*); anterior half of elytral ground moderately punctate (densely punctate in *Cymatodera grossa*); interstices 2.0 × the width of punctation (less than the width of punctation in *Cymatodera grossa*). Likewise, the male of *Cymatodera sinuosa* can be distinguished from males of other species sharing similar fasciae pattern and remaining congeners, by the unique combination of elongated, deeply incised, posteriorly elevated, and abruptly descended emargination on the sixth visible ventrite (Fig. 26).

#### Distribution.

The species is known from two localities: La Muralla National Park, situated in the department of Olancho, Honduras and El Boquerón National Park, in the Department of San Salvador, El Salvador.

#### Etymology.

The specific name comes from the Latin noun *sinuosa* (=sinuous), and refers to the winding character of the fasciae found on the elytral ground of this species.

### Cymatodera vittata

Burke sp. n.

urn:lsid:zoobank.org:act:7BC2D6CE-E909-4FB2-A162-5B7980ECA1F1

http://species-id.net/wiki/Cymatodera_vittata

Figs 4 , 13 , 27, 34 , 41, 48 , 55 , 58

#### Type material.

**Holotype:** male, Panamá, Provincia de Panamá, 8-10 km N El Llano, 24-V to 2- VI-1992, E. Giesbert, red handwritten label, holotype deposited in FSCA. **Paratypes:** 2 males, 6 females. 1 male: same data as holotype (USNM, 1); 2 females: Panamá, Provincia de Coclé, 4 km S El Valle, 2-VI-1981, E. Giesbert (KSUC, 1; JNRC, 1); 1 female: Panamá, Provincia de Coclé, 2 km W El Valle, 28-V-1981, E. Giesbert (JEWC, 1); 1 female: Panamá, Provincia de Coclé, Anton-El Valle, 880 m, 27-XII-1993, subtropical moist forest, beating vegetation, J. & E. Beierl (INBC, 1); 1 male: Panamá, Provincia de Panamá, El Llano-Carti, 9-I-1994, J. E. Wappes (JEWC, 1); 1 female: Panamá, Zona del Canal, 8 km NW Gamboa, (9°10.067'N, 79°45.017' W), 100 m, canopy fogging in *Luehea seemanni*, pyrethrin fog, 12-VII-1976, Montgomery and Lubin (WFBC, 1); 1 female: Panamá, Provincia de Panamá, Fuerte Kobbe, 20-I-1996, F. T. Hovore (WOPC, 1).

#### Description.

Size: TL= 7.8 mm, length of males 7.5 to 9.2 mm, length of females 6.5–7.8 mm, n = 9 (Fig. 4).

Color: head fuscous-brown; pronotum, mouthparts, mesosternum, metasternum and abdomen testaceous; elytral ground predominantly testaceous except median region pale-testaceous; legs mostly testaceous, except posterior half of femora brown. Each elytron with two pairs of black, irregularly marked maculae; the first adjacent to anterior margin, extending from second stria to humeral angle; the second located on first half of second fourth, more faintly marked than preceding pair, extending from second to eighth stria; a long, irregular, transversally marked, black fascia located on third fourth, in the form of a vitta, extending from suture to epipleuron, covering about one fourth of elytral length.

Head: HL= 1.3 mm, HW= 1.25 mm; length to width ratio: males average 1.06, females average 1.12; measured across eyes wider than pronotum; densely, coarsely punctate; surface rugose; vested with short, semirecumbent setae interspaced with some long, erect setae. Eyes rather small, subsinuate, longer than wide, moderately emarginate in front, somewhat bulging laterally, separated by approximately 3.5 eye-widths. Antennae extending beyond basal sixth of elytra; antennomeres 2-3 subequal in length; fourth antennomere 1.5 × longer than preceding antennomere; antennomeres 4-5 subequal in length; sixth antennomeres slightly shorter than fifth antennomere; antennomeres 6-10 subequal in length; antennomeres 2-5 slender; antennomeres 6-10 weakly serrate; last antennomere 1.5 × longer than tenth antennomere, subsinuate, flattened apically (Fig. 13).

**Figures 58–59.**
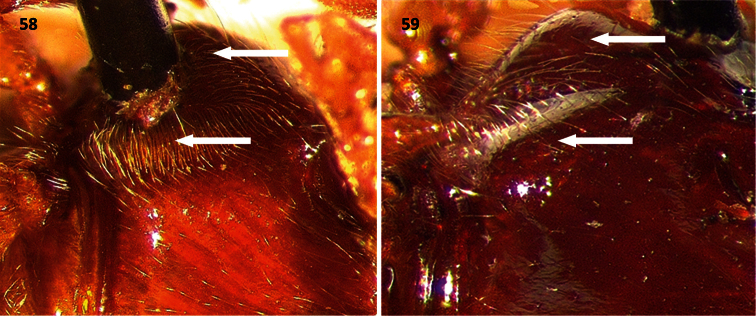
Metasterna of: **58** *Cymatodera vittata* (male) and **59** *Cymatodera rubida* (male). Arrows indicating dense vestiture in *Cymatodera vittata*, moderately developed in *Cymatodera rubida*.

Thorax: PL= 1.7 mm, PW= 1.2 mm; length to width ratio: males average 1.38, females average 1.46; pronotum widest at middle; sides constricted subapically, more strongly constricted behind middle; disc flat, rather impressed in front of middle; surface shiny; moderately, finely punctate; less densely punctate than head; somewhat covered with short, semirecumbent setae intermingled with long, erect setae; subbasal tumescences rather pronounced. Mesosternum moderately, coarsely punctate; vested with short, recumbent setae. Metasternum smooth; surface strongly convex, puncticulate laterally; median region with a sensory area consisting of rather dense, short, erect setae set on a rugose ground (Fig. 58). Scutellum semicircular; broader than long; clothed with short, recumbent setae.

Legs: covered with short, semirecumbent setae intermixed with some long, erect and suberect setae, vestiture become more densely arranged on distal half of tibiae; femora rugulose, feebly punctate; tibiae rugose, somewhat punctate.

Elytra: EL= 4.7 mm, EW= 1.85 mm; length to width ratio: males average 2.49, females average 2.38; anterior margin bisinuate, slightly broader than pronotum; sides subparallel; widest behind middle; base slightly wider than pronotum; humeri moderately indicated; disc subflattened above; apices rounded, dehiscent, covering sixth tergite; surface smooth, somewhat covered with short, erect and semierect setae interspaced with long, erect setae; sculpturing consisting of coarse, deep punctations arranged in striae that gradually reduce in size behind middle; interstices 2.0 × the width of punctation.

Abdomen: ventrites 1-5 rugulose; moderately, finely punctate; somewhat covered with short, recumbent setae combined with some long, erect setae. Fifth visible ventrite convex; lateral margins oblique; posterior margin broadly, deeply, arcuately emarginate (Fig. 27). Sixth visible ventrite slightly broader than long; surface concave, excavated, with a median carina initiating medially and reaching posterior margin, a pair of feebly pronounced anterolateral carinae extending from anterior margin to slightly beyond median region, not reaching posterior margin; lateral margins subparallel on first half, becoming oblique on second half; posterior margin deeply, semicircularly emarginate; hind angles, produced posteriorly, acuminate at apex; (Fig. 27). Fifth tergite shiny; surface somewhat convex; lateral margins feebly oblique; posterior margin narrowly, shallowly, arcuately emarginate; hind angles broadly rounded (Fig. 41). Sixth tergite subrectangular; rugulose; longer than broad; surface convex; posterior half ventrally recurved; lateral margins subparallel, becoming feebly oblique on second half; posterior margin broadly, deeply incised, triangularly emarginate; hind angles produced posteriorly, acuminate, ventrally folded (Fig. 41). Lateral margins of sixth tergite extending beyond sixth visible ventrite. Aedeagus 1.55 mm long, rather robust; ratio of length of paramere to whole tegmen 0.37: 1; parameres well developed, broad, subtriangular; obtuse distally, phallobase conspicuously wide; phallus with copulatory piece acuminated at apex; phallic plate with a reduced number of long denticles along dorsal margin; phallobasic apodeme slender distally; endophallic struts slender (Fig. 55).

#### Variation.

Females differ from male specimens as follows: antennomeres 5-10 weakly serrate, sixth visible ventrite subtriangular, lateral margins oblique, posterior margin broadly, very shallowly emarginate (Fig. 34); sixth tergite subtriangular, lateral margins oblique, posterior margin feebly notched, hind angles broadly rounded (Fig. 48). Additionally, female specimens lack the setiferous area found on the metasternal region of males (Fig. 58). Elytral ground color is rather variable in both sexes, ranging from flavous to ferrugineous. Maculae on anterior half of elytral ground irregularly impressed, ranging from strongly marked to absent. Posterior fascia color ranges from light brown to piceous. Leg color is also rather inconsistent, ranging from uniformly brown to bicolored. One male with anterolateral carinae of sixth visible ventrite more strongly pronounced. One male with surface of sixth ventrite feebly convex. Two females with posterior margin of sixth tergite truncate.

#### Differential diagnosis.

Its distinct elytral ground color, slender form, shape of terminal abdominal segments and male genitalia will readily separate this species from congeners. *Cymatodera vittata* appears especially similar to *Cymatodera rubida*, a sympatric species described below. Differences in antennal shape (Figs 13, 14) serve to separate this new species from its closest congener. *Cymatodera vittata* has the fourth antennomere conspicuously longer than preceding antennomere (antennomeres 3-4 about the same length in *Cymatodera rubida*); antennomeres 5-10 are somewhat longer and extend beyond basal sixth of elytral ground (antennomeres 5-10 are slightly shorter in *Cymatodera rubida* and do not extend beyond basal sixth of elytral ground). Additionally, the distinctive metasternal sensory area found in the male of *Cymatodera vittata* is poorly developed in males of *Cymatodera rubida* (Figs 58, 59). Differences in terminalia (Figs 27, 28, 41, 42) and male genitalia (Figs 55, 56) also distinguish this new species from *Cymatodera rubida*.

#### Distribution.

Known from three localities in Panamá: El Llano, Panamá Province; 4 km south of Gamboa, Colón Province; and 4 km south of El Valle, Coclé Province.

#### Etymology.

The specific epithet comes from the Latin noun *vitta* (=band), and refers to the posterior fascia on the elytral ground of this new species.

### Cymatodera rubida

Burke sp. n.

urn:lsid:zoobank.org:act:398AFCAB-A1D8-49DD-8809-6B40230CED6F

http://species-id.net/wiki/Cymatodera_rubida

Figs 5 , 14 , 28, 35 , 42, 49 , 56 , 59

#### Type material.

**Holotype:** male, Panamá, Zona del Canal, Fuerte Kobbe, 125 m, X-30-1980, H. Stockwell, red handwritten label, holotype deposited in INBC. **Paratypes:** 8 males, 4 females. 1 male and 2 females: Panamá, Provincia de Colón, 4.5 km NE Palenque, 25-II to 4-III-1992, E. Giesbert (USNM, 1; WFBM, 1; KSUC, 1); 1 male: Panamá, Provincia de Panamá, Cerro Azul, 14 to 25-I-1993, F. T. Hovore (KSUC, 1); 1 female: Panamá, Provincia de Panamá, km 8-11 carretera El Llano-Carti, 330 m, 24-V to 2-VI-1992, J. E. Wappes (JEWC, 1); 1 female: Panamá, Provincia de Panamá, 12 km NE El Llano, 23-I-1993, F. T. Hovore (FSCA, 1); 1 male: Panamá, Provincia de Panamá, Cerro Azul/Jefe, 17-I-1996, F. T. Hovore (JNRC, 1); 3 males: Panamá, Provincia de Panamá, NW of Tocumén, Cerro Azul, 12.8 km N C[arretera] Panamericana, 650 m, 18-XII-1993, tropical dry forest, J. & E. Beierl (JNRC, 2; WOPC, 1); 1 male: Panamá, Zona del Canal, 8 km NW Gamboa, (9°10.067'N, 79°45.017'W), 100 m, canopy fogging in *Luehea seemanni*, pyrethrin fog, 12-VII-1976, Montgomery and Lubin (USNM, 1); 1 male: Panamá, Provincia de Panamá, Ancón Hill, 22-II-1983, D. Yanega (SEMC, 1).

#### Description.

Size: TL= 7.7 mm, length of males 7 - 9.2 mm, length of females 6.8 - 9.8 mm, n = 13 (Fig. 5).

Color: head, pronotum, prosternum, mesosternum, metasternum, and abdomen ferrugineous; elytral ground dark-testaceous; legs, antennae and mouthparts testaceous; sixth visible ventrite light brown, slightly lighter than remaining visible ventrites (Fig. 28). Each elytron with two irregular fasciae; first slender, brown, extending from elytral suture to humeral angle, surrounding scutellum; second fascia broad, fuscous, slightly darker than preceding band, in the form of a vitta, located on third fourth, extending from elytral suture to ninth stria, not reaching epipleuron, covering about one fourth of elytral length.

Head: HL= 1.2 mm, HW= 1.4 mm; length to width ratio: males average 0.91, females average 1.02; measured across eyes wider than pronotum; densely, moderately coarsely punctate; surface rugose; covered with short, recumbent setae intermixed with less abundant, long, erect setae that become more densely arranged behind eyes; frons moderately bi-impressed. Eyes rather small, subsinuate, longer than wide, feebly emarginate in front, somewhat bulging laterally, separated by approximately 3.5 eye-widths. Antennae extending to basal sixth of elytra; antennomeres 2-3 subequal in length; fourth antennomere inconspicuously longer than third antennomere; antennomeres 4-10 subequal in length; antennomeres 2-5 slender; antennomeres 6-10 weakly serrate; last antennomere subsinuate, flattened apically, about 1.5 × longer than tenth antennomere (Fig. 14).

Thorax: PL= 1.7 mm, PW= 1.2 mm; length to width ratio: males average 1.39, females average 1.33; pronotum widest at middle; sides constricted subapically, more constricted behind middle; surface somewhat rugose; disc flat, somewhat impressed in front of middle; moderately, finely punctate; less densely, deeply punctate than head; clothed with short, semirecumbent setae intermingled with long, erect setae; subbasal tumescences pronounced. Mesosternum rugulose, coarsely punctate. Metasternum rugose, moderately, coarsely punctate; somewhat clothed with short, recumbent setae; absence of sensory area (Fig. 59). Scutellum subquadrate; broader than long; covered with short, recumbent setae.

Legs: vested with short, recumbent setae, intermixed with longer semierect setae, vestiture becomes more abundant on internal face of tibiae; femora rugulose, finely punctate; tibiae rugose, moderately, coarsely punctate.

Elytra: EL= 4.5 mm, EW= 2.0 mm; length to width ratio: males average 2.18, females average 2.12; anterior margin bisinuate, slightly broader than pronotum; sides subparallel, inconspicuously wider behind middle; humeri moderately indicated; apices rounded, dehiscent; covering sixth tergite; surface shiny, vested with short, recumbent setae interspaced with some long, erect setae; sculpturing consisting of coarse, deep punctations arranged in striae that gradually reduce in size behind middle; interstices smooth, 2.0 × the width of punctuation.

Abdomen: ventrites 1-4 moderately, finely punctate; smooth; somewhat vested with fine, short, recumbent setae interspaced with few long, erect setae; hind margins truncate. Fifth visible ventrite smooth; surface convex; lateral margins oblique; posterior margin broadly, deeply, arcuately emarginate (Fig. 28). Sixth visible ventrite subquadrate; broader than long; surface slightly convex; rugose; moderately, finely punctate, less densely punctate than preceding ventrite; lateral margins oblique; posterior margin broadly, deeply, arcuately emarginate; hind angles arcuate (Fig. 28). Fifth tergite rugulose; surface convex; puncticulate; lateral margins feebly oblique; posterior margin shallowly, broadly, arcuately emarginate. Sixth tergite subtriangular; surface convex; rugulose; broader than long; lateral margins oblique; posterior margin rounded, with a median, shallow, broad, triangular emargination; hind angles rounded (Fig. 42). Posterior margin of sixth tergite extending slightly beyond apical margin of sixth visible ventrite. Aedeagus 1.4 mm long, slender; ratio of length of paramere to whole tegmen 0.39: 1; parameres narrow, parallel, obtuse distally; phallus with copulatory piece somewhat tapered at apex; phallic plate with numerous fine, small denticles along dorsal margin; phallobasic apodeme rather broad, not dilated distally; endophallic struts slender (Fig. 56).

#### Variation.

Female specimens have the sixth visible ventrite subquadrate, rugulose, surface strongly convex, lateral margins oblique, posterior margin truncate (Fig. 35); sixth tergite campanulate, rugulose, surface convex, lateral margins oblique, becoming parallel on last third, then abruptly expanding before apex, posterior margin broadly, deeply notched (Fig. 49); posterior margin of sixth tergite extending beyond apical margin of sixth visible ventrite, posterolateral margins of sixth visible ventrite projecting laterally beyond sixth tergite (Figs 35, 49). Elytral marking on anterior half ranges from strongly impressed to absent. Posterior fasciae color is also rather variable, ranging from light brown to piceous. Leg color ranges from uniformly brown to bicolored. One male with posterior margin of sixth ventrite slightly less incised than remaining male specimens in the type series.

#### Differential diagnosis.

*Cymatodera rubida* is separable from congeners based on its size, shape, elytral markings, terminal abdominal segments and male genitalia. Most similar to *Cymatodera vittata*, but differs from this by having the fourth antennomere about the same size as third antennomere (Fig. 14), rather than 1.5 × longer (Fig. 13). Unlike *Cymatodera vittata*, antennomeres 5-10 of *Cymatodera rubida* are rather short; hence, antennae do not extend beyond basal sixth of elytral ground. Furthermore, male specimens of *Cymatodera rubida* do not display the moderately dense vestiture found on the metasternal area of males of *Cymatodera vittata* (Figs 58–59). Conspicuous differences in terminal abdominal segments (Figs 27, 28, 41, 42) and male genitalia (Fig. 55, 56) also distinguish this new species from *Cymatodera vittata*.

#### Distribution.

Known from five localities in Panamá: the Canal Zone, 5 km south of Gamboa; Fuerte Kobbe; the mountainous region of Cerro Azul; the surroundings of Palenque; and El Llano-Carti road, 12 km north of El Llano.

#### Etymology.

The specific epithet comes from the Latin noun *rubida* (=red), this adjective indicates the general reddish color of this new species.

### Cymatodera limatula

Burke sp. n.

urn:lsid:zoobank.org:act:660A0D71-FDA1-4FA0-BDD6-29CDCE5740F4

http://species-id.net/wiki/Cymatodera_limatula

Figs 6 , 15 , 29, 36 , 57 , 60, 61, 62

#### Type material.

**Holotype:** male, México, Chiapas, El Aguacero, 680 m, at light, 17-VI-1990, R. A. Cunningham, red handwritten label, holotype deposited in CNIN. **Paratypes:** 2 males, 1 female. 1 male: same data as holotype (JNRC, 1); 1 female: México, Chiapas, El Chorreadero, 10 km W Chiapa de Corzo, 24-VI-1987, E. Giesbert (USNM, 1); 1 male: México, Chiapas, C[arretera] 190, 17 km W Tuxtla Gutierrez, Alt. 1000 m, 21 to 25-VI-1987, E. Giesbert (KSUC, 1).

#### Description.

TL= 9.8 mm, length of males 10.2–11 mm, length of female 9.4 mm, n = 4 (Fig. 6).

**Figures 60–62.**
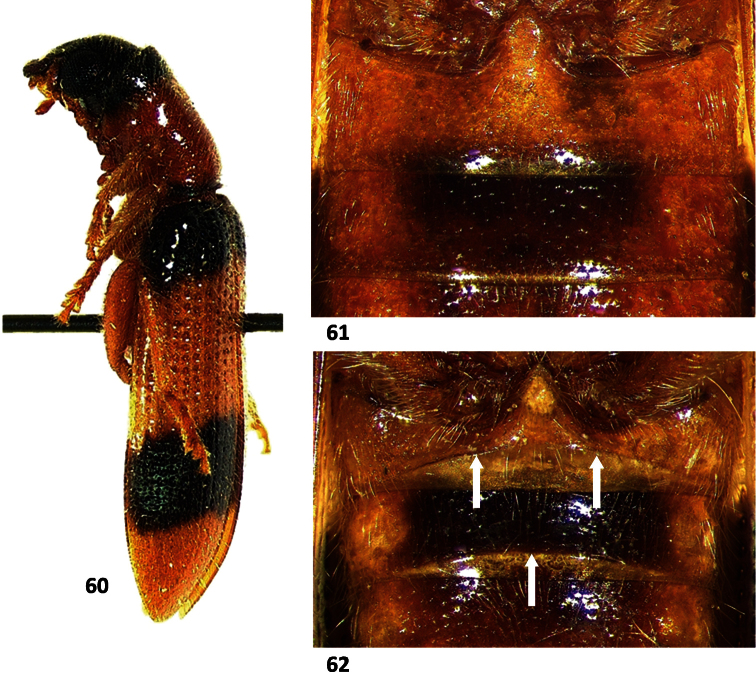
*Cymatodera limatula*: **60** Lateral view **61** First and second visible ventrites of female **62** First and second visible ventrites of male, arrows indicating transverse carinae.

Color: head predominantly black (Fig. 60), except gula and submentum ferrugineous; pronotum and mesosternum light ferrugineous; elytra, metasternum, legs, mouthparts and antennae testaceous; abdomen testaceous, except sixth visible ventrite pale testaceous (Fig. 29), and median region of ventrites 2-3 irregularly fuscous (Fig. 62); vestiture uniformly pale; a transversally directed, black fascia on anterior margin of pronotum covering about one fifth of pronotal disc, and two irregular, small, black maculae located on lateral area of pronotum adjacent to posterior margin. Each elytron with two broad, somewhat irregular, transversally directed, black fasciae extending from suture to epipleuron; first located on humeral region, covering about one sixth of elytral ground; second behind middle, somewhat longer than preceding band, covering approximately one fifth of elytral ground.

Head: HL= 1.3 mm, HW= 1.5 mm; length to width ratio: males average 0.92, female 0.86; measured across eyes wider than pronotum; surface rugose; frons feebly bi-impressed; moderately, rather coarsely punctate; clothed with short, recumbent setae intermixed with some long, semierect and erect setae. Eyes medium-sized, rather rounded, inconspicuously longer than wide, feebly emarginate in front, somewhat bulging laterally, separated by approximately 3 eye-widths. Antennae reaching humeral angles; antennomeres 2-3 subcylindrical, slender; antennomeres 4-10 strongly serrate, longer than broad; third antennomere somewhat longer than second antennomere; fifth antennomere slightly shorter than fourth antennomere; antennomeres 5-10 subequal in length; last antennomere 2.5 × longer than tenth antennomere (Fig. 15).

Thorax: PL= 2.1 mm, PW= 1.15 mm; length to width ratio: males average 1.85, female 1.75; pronotum rugose; widest at middle; middle slightly wider than front margin; sides constricted subapically, more strongly constricted behind middle; disc flat, feebly impressed in front of middle; subbasal tumescence pronounced; vested with short and long semirecumbent setae interspaced with some erect setae; surface moderately, finely punctate. Mesosternum rugose; moderately, coarsely punctate; somewhat clothed with long, semirecumbent setae.

Metasternum shiny; surface convex, puncticulate; covered with long, semirecumbent setae. Scutellum subquadrate; wider than long; notched medially.

Legs: clothed with long, erect setae and some short, recumbent setae that become more abundant on posterior half of tibiae; femora shiny, finely, indistinctly punctate, transversely rugose; tibiae coarsely, densely punctate, longitudinally rugose.

Elytra: EL= 6.1 mm, EW= 2.8 mm; length to width ratio: males average 2.15, female 2.18; anterior margin bisinuate, wider than pronotum; disc smooth, flattened above; humeri indicated; sides subparallel, widest on third fourth; apices weakly dehiscent, rounded, covering sixth tergite; elytral declivity somewhat procurved, clothed with short, semirecumbent setae intermingled with long, erect, less densely arranged setae; sculpturing consisting of coarse punctations arranged in striae that gradually reduce in size behind middle; interstices smooth, about 1.5 × the width of punctation.

Abdomen: ventrites 1-5 rugose; moderately, coarsely punctate; somewhat clothed with short, recumbent setae interspaced with some long, erect setae. First visible ventrite strongly convex; posterior margin elevated, with a transverse carina that initiates next to hind angles and produces a broad, deep, arcuate emargination (Fig. 62). Second visible ventrite rather convex; posterior margin elevated, moderately, arcuately emarginate (Fig. 62). Ventrites 3-4 feebly convex; hind margins truncate. Fifth visible ventrite somewhat convex; lateral margins oblique; posterior margin broadly, rather deeply, arcuately emarginate; hind angles narrowly rounded (Fig. 29). Sixth visible ventrite subquadrate; rugulose; surface feebly convex; broader than long; puncticulate; second half with a median tumescence; lateral margins oblique; posterior margin broadly, shallowly, triangularly emarginate; hind angles rounded (Fig. 29). Fifth tergite rugulose; surface weakly convex; finely punctate; posterior margin narrowly, shallowly, arcuately emarginate. Sixth tergite subtriangular; rugulose; surface somewhat convex; moderately punctate; lateral margins strongly oblique, narrowing apically, producing a constricted, rather acuminate posterior margin. Sixth tergite extending slightly beyond the apical margin of sixth visible ventrite. Aedeagus 1.85 mm long, rather slender; ratio of length of paramere to whole tegmen 0.28: 1; parameres feebly developed, pointed at apex, phallobase wide; phallus with copulatory piece acuminated distally, phallic plate without an internal row of denticles at dorsal margin, with fine granular structures on posterior area; phallobasic apodeme and endophallic struts elongate, slender (Fig. 57).

#### Variation.

The only female in the type series differs from the male by having the first visible ventrite moderately longer and the posterior margin of ventrites 1-2 truncate (Fig. 61). Moreover, abdominal differences are as follows: fifth visible ventrite rugose, lateral margins oblique, posterior margin truncate (Fig. 36); sixth visible ventrite rugulose, semicircular, surface feebly convex, broader than long, lateral and posterior margins broadly rounded (Fig. 36); fifth tergite rugulose, lateral margins oblique, posterior margin truncate; sixth tergite rugulose, broader than long, surface inconspicuously convex, lateral and posterior margins strongly oblique, slightly acuminate posteriorly, almost semicircular. Posterior margin of sixth tergite extending slightly beyond sixth visible ventrite. One male paratype does not possess the irregular, small, black maculae located on the posterolateral area of pronotum.

#### Differential diagnosis.

The distinctive coloration and elytral markings of this species will serve to separate it from congeners. No other species in the *Cymatodera* group has the distinctive piceus fasciae on the pronotum and elytral ground, and the predominantly black color on the head (Figs 6, 60). In addition, shape of terminal abdominal segments (Figs 29, 36), strongly serrate condition of antennomeres 4-10, shape of last antennomere (Fig. 17), and male genitalia (Fig. 57) are characters that serve to separate *Cymatodera limatula* from remaining *Cymatodera* species.

#### Distribution.

This species is known from three localities in the state of Chiapas, Mexico: El Aguacero, in the periphery of Tuxtla Gutierrez; El Chorreadero, 8 km northeast of Chiapa de Corzo; and on highway 190, 17 km west of Tuxtla Gutierrez.

#### Etymology.

The specific epithet comes from the Latin noun *limatula* (=distinctive), and makes allusion to the characteristic color pattern of this new species.

### Cymatodera obliquefasciata

Schaeffer, 1904

http://species-id.net/wiki/Cymatodera_obliquefasciata

Figs 7 , 16 , 23 , 30, 37 , 43, 50

Cymatodera obliquefasciata Schaeffer 1904. 215. TX: Esperanza Ranch, Brownsville [Cameron Co.]; TX. Lectotype designated by Chapin, 1949: 8. (Lecotype deposited in: USNM; sex of lectotype: male).

#### Material examined.

(5 males, 2 females). 2 males: Texas, Hidalgo Co., Bentsen Rio Grande State Park, 16-VI-1974, G. H. Nelsen (FSCA); 3 males and 1 females: USA, Texas, Hidalgo Co., LRGVNWR, MacManus unit, (26°3.228'N, 98°2.9922'W), 5-V-1994, UV light in ebony-guayacan association, J. King and E. Riley (TAMU); 1 female: USA, Texas, Cameron Co. Sabal Palm Grove Reservation site 1, (25°50.8794'N, 97°25.1286'W), UV light in palm forest, J. King and E. Riley (TAMU).

#### Diagnosis.

*Cymatodera obliquefasciata* can be distinguished from *Cymatodera rosalinae* based on antennal differences. *Cymatodera obliquefasciata* presents the antennomeres 1-3 slender and the antennomeres 4-10 strongly serrate (Fig. 16). Conversely, *Cymatodera rosalinae* has the antennomeres 2–5 slender and the anntenomeres 5–10 feebly serrate in males (Fig. 9) and moderately serrate in females (Fig. 10). Differences in male (Figs 24, 30) and female (Figs 31, 37) terminalia also serve to readily separate these species.

#### Distribution.

*Cymatodera obliquefasciata* is known from Texas (Opitz, 2010; Wolcott, 1947) and New Mexico (Wolcott, 1947). Barr, in his most recent catalogue (1999, unpublished), points out that the geographical distribution of this species extends southward into north Mexico, yet, no material from Mexico was examined.

## Supplementary Material

XML Treatment for Cymatodera

XML Treatment for Cymatodera rosalinae

XML Treatment for Cymatodera capax

XML Treatment for Cymatodera sinuosa

XML Treatment for Cymatodera vittata

XML Treatment for Cymatodera rubida

XML Treatment for Cymatodera limatula

XML Treatment for Cymatodera obliquefasciata
